# Temporal Evolution and Dissociation of Neuroendocrine Stress Inflammatory Responses Neurological Dysfunction and Multiorgan Injury Following Experimental Polytrauma in Mice

**DOI:** 10.3390/biology15191788

**Published:** 2026-10-08

**Authors:** Bing Liu, Fang Xie, Ke Dong, Yumeng Wang, Ling Zhang, Yun Zhao, Xue Wang, Zhaowei Sun, Shenghui Zhang, Jiani Jing, Ling-Jia Qian, Jianning Zhang

**Affiliations:** 1 The Academy of Military Medical Sciences, Beijing 100850, China; 2 Department of Neurosurgery, First Medical Center of the PLA General Hospital, Beijing 100853, China

**Keywords:** polytrauma, neuroendocrine stress, corticosterone, neuroinflammation, neurological dysfunction, multiorgan injury, temporal dynamics

## Abstract

Polytrauma induces neuroendocrine, inflammatory, neurological, and multiorgan responses that may follow distinct temporal trajectories. We characterized these changes during the first 7 days after experimental head–thorax–abdomen polytrauma in male C57BL/6 mice. Animals underwent polytrauma or Sham procedures and were evaluated at 0 h, 3 h, 6 h, 12 h, 24 h, 3 d and 7 d. Neurological and behavioral outcomes, brain water content, serum and injured-brain neuroendocrine and inflammatory mediators, and brain, lung, and liver histopathology were assessed. Polytrauma caused persistent neurological and locomotor impairment and increased brain water content. Corticosterone rose rapidly during the early phase and largely normalized by 12 h, whereas norepinephrine in injured brain tissue increased at 3–7 d. Pro- and anti-inflammatory mediators showed distinct systemic and cerebral temporal profiles. Histology demonstrated time-dependent brain, lung, and liver injury with evolving extracellular matrix remodeling. Thus, experimental polytrauma produces temporally dissociated responses characterized by early glucocorticoid activation, persistent neurological and inflammatory abnormalities, delayed cerebral noradrenergic changes, and progressive multiorgan remodeling, supporting phase-specific pathophysiology after injury.

## Simple Summary

Polytrauma involving the head, thorax, and abdomen induces a complex biological response that evolves over time rather than occurring simultaneously across different organs and physiological systems. In this study, polytrauma triggered a rapid glucocorticoid response, whereas norepinephrine elevation in injured brain tissue occurred later. In contrast, neurological impairment, cerebral edema, inflammatory responses, and pathological changes in multiple organs persisted beyond the initial endocrine stress phase. These findings demonstrate a clear temporal dissociation between systemic stress responses, cerebral neuroendocrine changes, neurological dysfunction, inflammation, and multiorgan injury. The identification of distinct early, intermediate, and subacute response phases highlights the dynamic nature of polytrauma and suggests that therapeutic interventions may need to be tailored to specific stages after injury rather than applied uniformly across the entire post-traumatic period.

## 1. Introduction

Polytrauma is characterized by simultaneous or sequential injuries involving multiple anatomical regions and organ systems and is associated with substantial morbidity and mortality [1,2,3,4]. Beyond the primary mechanical damage, severe trauma initiates a complex systemic response involving neuroendocrine activation, immune dysregulation, endothelial dysfunction, metabolic disturbances, and secondary injury in both the central nervous system and peripheral organs. The magnitude and duration of these responses vary according to injury burden, anatomical distribution, and the time elapsed after trauma, making polytrauma a dynamic biological process rather than a static disease state [2,3,4,5,6].

The neuroendocrine stress response represents one of the earliest systemic adaptations to severe injury. Glucocorticoids rise rapidly after trauma and contribute to cardiovascular, metabolic, and immune homeostasis during the immediate post-injury period [7]. Catecholaminergic signaling is also involved in the response to traumatic stress, but circulating and tissue catecholamine concentrations may not evolve in parallel with glucocorticoid responses [8,9,10]. Importantly, measurements of circulating mediators alone may not capture local changes within injured tissue, particularly in the brain, where neuroendocrine and inflammatory signals can be modified by blood–brain barrier disruption, neuronal injury, glial activation, and regional neurotransmitter metabolism [11,12].

Inflammation is another major component of the response to polytrauma. Tissue disruption triggers the release of damage-associated molecular patterns and rapidly activates innate immune pathways, leading to production of pro-inflammatory cytokines such as interleukin IL-1β, IL-6 and tumor necrosis factor-α (TNF-α) [13,14,15,16]. These mediators can contribute to cerebral edema, neuronal dysfunction, pulmonary injury, hepatic injury, and systemic immune disturbances [13,17,18,19]. At the same time, anti-inflammatory and immune-regulatory mediators, including IL-4, IL-10 and transforming growth factor-β (TGF-β), are recruited to limit excessive inflammation and facilitate tissue repair [14,17,20]. The balance between these pro-inflammatory and counter-regulatory components is therefore likely to change over time rather than remain constant [16,20,21].

The temporal dimension is especially important in polytrauma because biological processes that dominate the first hours after injury may not remain dominant several days later. A rapid endocrine stress response may subside while neurological dysfunction, local inflammation, and tissue remodeling continue. Similarly, systemic inflammatory markers may normalize even [2,3,4,5,6] though organ-specific inflammation persists. Failure to account for these temporal differences may obscure biologically meaningful therapeutic windows and may partly explain why interventions targeting a single pathway at a single time point often show limited efficacy [5,10,18,21,22,23].

Most experimental trauma studies focus on a single injured organ, a restricted set of biomarkers, or a narrow observation window. Integrative studies that simultaneously track neurological function, behavior, neuroendocrine mediators, systemic and cerebral inflammatory responses, and pathology in multiple organs across acute and subacute phases remain comparatively limited [2,5,24,25]. The present study therefore used an experimental head–thorax–abdomen polytrauma model to define the temporal evolution of neurological dysfunction, cerebral edema, neuroendocrine stress mediators, systemic and injured-brain inflammation, and brain, lung, and liver pathology from immediately after injury through day 7. We hypothesized that these biological responses would exhibit distinct temporal trajectories, with an early glucocorticoid-dominant stress phase followed by more persistent inflammatory, neurological, and tissue-remodeling responses.

## 2. Materials and Methods

### 2.1. Animals

Male C57BL/6 mice aged 6–8 weeks and weighing 20–22 g were used. A restricted young age range was selected to minimize age-related biological variability during characterization of the temporal response to polytrauma. Animals were obtained from HuaFuKang Company (Beijing, China). Mice were housed under controlled laboratory conditions at 22 ± 2 °C and 50 ± 10% humidity on a 12 h light/dark cycle with free access to food and water. Animals were acclimatized before experimentation. All procedures were approved by the Institutional Animal Care and Use Committee of the Academy of Military Medicine Sciences (Permit No: IACUC-DWZX-2022-534) and were conducted in accordance with institutional animal-care. Guidelines and the ARRIVE recommendations [26].

### 2.2. Experimental Design and Sample Size

**Figure 1:**
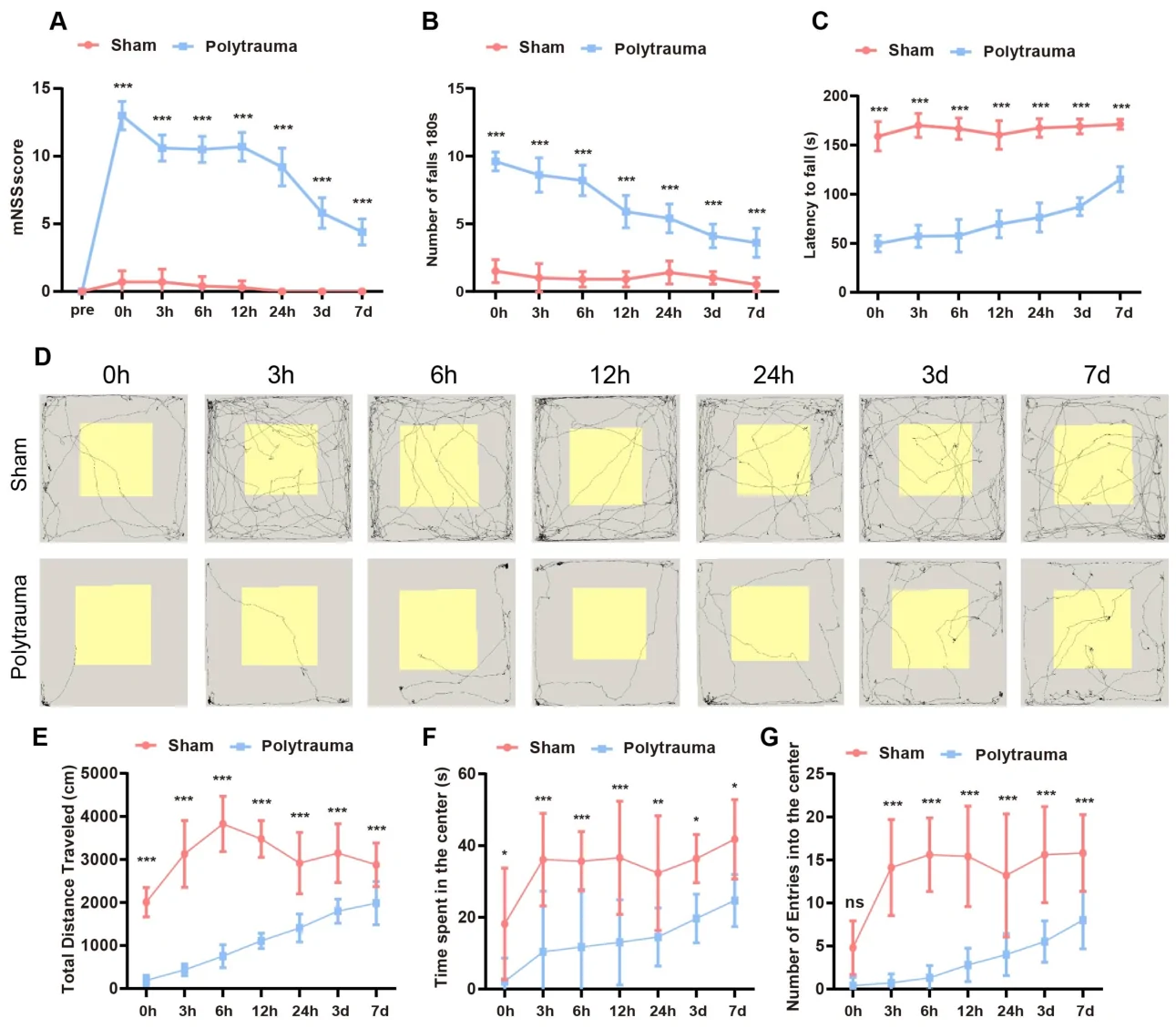
Experimental design and polytrauma-induced neurological, motor, and exploratory dysfunction. (**A**) Modified neurological severity score (mNSS). (**B**) Number of falls during a 180-s rotarod test. (**C**) Rotarod latency to fall. (**D**) Representative open-field movement trajectories. (**E**) Total distance traveled. (**F**) Time spent in the center. (**G**) Number of entries into the center. Longitudinal behavioral data: n = 10 mice per group. For behavioral measurements, the 0 h assessment was performed after recovery from anesthesia; for terminal biological sampling, 0 h refers to immediately after completion of the polytrauma procedure. Data are mean ± SD. Statistical annotations shown in the current figure set represent Sham-versus-polytrauma comparisons: * *p* < 0.05, ** *p* < 0.01, *** *p* < 0.001; ns—not significant.

Mice were assigned to Sham and polytrauma groups. Post-injury assessments were performed at seven time points: 0 h, 3 h, 6 h, 12 h, 24 h, 3 d and 7 d. Seven post-injury observation points were designated as 0 h, 3 h, 6 h, 12 h, 24 h, 3 d, and 7 d. For terminal serum and tissue collection, the 0 h time point corresponded to sampling immediately after completion of the polytrauma procedure. For longitudinal behavioral testing, the assessment designated as 0 h was performed approximately 15 min after injury, after the mice had recovered sufficiently from anesthesia to regain spontaneous righting and locomotor activity. Therefore, the operational timing of the 0 h assessment differed between behavioral testing and terminal sampling. Longitudinal behavioral measurements comprising the mNSS, rotarod test, and open-field test were performed within the same experimental cohort (n = 10 mice per group). Brain water content was assessed in 3 independent mice per group at each time point (n = 3). Histopathological examinations were performed using tissues from polytrauma mice at each time point, together with a single Sham histology cohort used as the common reference across all post-injury time points. Enzyme-linked immunosorbent assay (ELISA) measurements in serum and injured brain tissue were performed using samples from 6 independent mice per group at each time point (n = 6). The experimental timeline is shown in Figure 1A.

### 2.3. Experimental Head–Thorax–Abdomen Polytrauma Model

Experimental polytrauma was induced by combining traumatic injuries to the brain, thorax, and abdomen. Tribromoethanol (Meilunbio, Dalian, China, MA0478) was administered by intraperitoneal injection for anesthesia at a dose of 30 μL/g. Injuries were sequentially inflicted in the order of controlled cortical impact-induced traumatic brain injury, blunt thoracic impact, and abdominal mechanical injury without intervening time intervals.

#### 2.3.1. Controlled Cortical Impact-Induced Traumatic Brain Injury

For traumatic brain injury, anesthetized mice were placed in the prone position. A midline scalp incision was made to expose the skull. A circular 4 mm craniotomy was created using a dental drill at a site 2 mm posterior to bregma and 1.5 mm lateral to the left of the sagittal suture. Controlled cortical impact was delivered at an impact velocity of 4.0 m/s, an impact depth of 1.5 mm, and a dwell time of 250 ms. The incision was then closed according to the experimental protocol [27,28].

#### 2.3.2. Blunt Thoracic Impact Injury

For thoracic trauma, anesthetized mice were positioned supine. The impact site was localized approximately 3 mm superior and 3 mm to the right of the manubrium using laser-guided positioning. Blunt thoracic injury was produced by free fall of a steel ball weighing 20 g and measuring 17 mm in diameter from a height of 40 cm onto the predefined thoracic site [29].

#### 2.3.3. Mechanical Hepatic Injury

For abdominal trauma, anesthetized mice were placed supine. A 1.5 cm midline laparotomy was performed immediately inferior to the xiphoid process. One liver lobe was gently exteriorized using a sterile cotton swab, and three approximately 5-mm-long incisions were rapidly made in the exposed lobe using surgical scissors to induce standardized mechanical hepatic injury. The liver was returned to the abdominal cavity and the incision was closed.

### 2.4. Sham Procedure

Sham mice received the same Avertin anesthesia by intraperitoneal injection and underwent scalp and abdominal skin incisions only. No craniotomy, cortical impact, thoracic impact, opening of the abdominal cavity, hepatic incision, or other organ injury was produced.

For histopathological comparisons, a single Sham group was used as the common reference rather than separate Sham cohorts at each post-injury time point. This design was selected because Sham animals underwent only superficial scalp and abdominal skin incisions and did not receive craniotomy, cortical impact, thoracic impact, laparotomy, hepatic incision, or any direct organ injury. Accordingly, repeated histological sampling of separate Sham cohorts across all seven time points was not expected to provide additional information regarding trauma-induced pathological changes. The single Sham histology cohort was therefore compared with the polytrauma groups at 0 h, 3 h, 6 h, 12 h, 24 h, 3 d and 7 d, while also minimizing unnecessary animal use.

### 2.5. Modified Neurological Severity Score

Neurological function was evaluated using the modified neurological severity score (mNSS) in 10 mice per group. The same animals were longitudinally assessed before injury and at the designated post-injury time points. The mNSS integrates motor, sensory, balance, and reflex functions, with higher scores indicating more severe neurological impairment [30].

### 2.6. Rotarod Test

Motor coordination and balance were assessed using the rotarod test in 10 mice per group [31]. Animals were tested before injury and at the designated post-injury time points. Latency to fall and the number of falls during a 180-s observation period were recorded. The rotarod was started at 4 rpm and accelerated at 4 rpm/min throughout the test.

### 2.7. Open-Field Test

Spontaneous locomotor activity and exploratory behavior were assessed in a 50 cm × 50 cm open-field arena [32]. Each mouse was individually placed in the arena and allowed to explore freely for 5 min. Movement was recorded and analyzed using the Noldus EthoVision XT 16 system from Noldus Information Technology BV, based in Wageningen, The Netherlands. Total distance traveled, time spent in the center, and number of center entries were quantified. The arena was cleaned between trials to minimize residual olfactory cues.

### 2.8. Brain Water Content

Brain water content was assessed in 3 mice per group at each time point as an index of cerebral edema using the wet–dry method [33]. Fresh brain tissue was weighed immediately after collection to obtain the wet weight and then dried at 65 °C for 72 h until a constant dry weight was reached. Brain water content was calculated as [(wet weight − dry weight)/wet weight] × 100%.

### 2.9. Sample Collection

At the designated post-injury time points, mice assigned to terminal sampling were anesthetized with Tribromoethanol administered by intraperitoneal injection at a dose of 30 μL/g. Peripheral blood was collected by orbital enucleation and allowed to clot at room temperature for 2 h. The samples were then centrifuged at 1000× *g*, and the resulting supernatant was collected as serum for subsequent analyses. Serum was stored at −80 °C until analysis. Brain tissue surrounding the injured cortical region was rapidly dissected. Tissue intended for ELISA was homogenized and centrifuged, and the supernatant was collected. Total protein concentration was determined using BCA. Separate brain, lung, and liver specimens were collected for histological analysis and fixed in 4% paraformaldehyde.

### 2.10. Measurement of Corticosterone and Norepinephrine

Corticosterone and norepinephrine concentrations were measured in peripheral serum and homogenates from injured brain tissue using commercially available ELISA kits according to the manufacturers’ instructions. Norepinephrine was measured using a kit from Elabscience Biotechnology Co., Ltd. (Wuhan, China, E-EL-0047). The concentration of corticosterone in mouse serum was determined using an enzyme-linked immunosorbent assay kit (CSB-E07969m, CUSABIO, Houston, TX, USA, https://www.cusabio.com/) according to the manufacturer’s instructions. Serum concentrations were expressed as ng/mL, and injured-brain concentrations were normalized to total protein content and expressed as ng/mg total protein. Each analysis used 6 biological replicates per group at each time point (n = 6).

### 2.11. Measurement of Inflammatory and Immune-Regulatory Cytokines

Concentrations of IL-1β, IL-6, TNF-α, IL-4, IL-10 and TGF-β in peripheral serum and injured brain tissue homogenates were determined using enzyme-linked immunosorbent assay kits (CSB-E08054m, CSB-E04639m, CSB-E04741m, CSB-E04634m-IS, CSB-E04594m-IS, CSB-E04726m-IS, CUSABIO, https://www.cusabio.com/) according to the manufacturer’s instructions. Assays were performed according to the manufacturers’ protocols. Serum cytokine concentrations were expressed as pg/mL. Concentrations in injured brain tissue were normalized to total protein and expressed as pg/mg total protein. Each analysis included 6 biological replicates per group at each time point (n = 6).

### 2.12. Histopathological Assessment of the Brain

Brain specimens encompassing the traumatic injury region were fixed in 4% paraformaldehyde, paraffin embedded and sectioned at 5 μm. Hematoxylin–eosin (HE) staining was used to assess tissue edema, neuronal degeneration, nuclear pyknosis, cytoplasmic eosinophilia, hemorrhagic and structural disruption and parenchymal disorganization. Adjacent sections were subjected to Nissl staining with 0.1% cresyl violet to evaluate neuronal structural integrity and Nissl substance in the injured region. Histopathological analyses were performed using tissues from polytrauma mice at each time point and tissues from the single Sham histology cohort as the common reference. Histopathological analyses were performed using tissues from three mice per group at each polytrauma time point (n = 3), with the single Sham histology cohort serving as the common reference. The HE and Nissl analyses were primarily qualitative and were used to characterize the temporal pattern of tissue injury; formal histopathological scoring or morphometric quantification was not performed.

### 2.13. Histopathological Assessment of the Lung and Liver

Lung and liver tissues were fixed in 4% paraformaldehyde, paraffin embedded, and sectioned at 5 μm. Pulmonary HE sections were examined for alveolar structural disruption, septal thickening, inflammatory-cell accumulation, edema, and hemorrhagic changes. Hepatic HE sections were examined for hepatocellular swelling, parenchymal disorganization, sinusoidal congestion, inflammatory-cell infiltration and focal necrotic changes. Histological analyses were performed using tissues from polytrauma mice at each time point and tissues from the single Sham histology cohort as the common reference. Lung and liver histopathological analyses were performed using three mice per group (n = 3). The analyses were qualitative and focused on temporal changes in tissue architecture and injury-associated features.

### 2.14. Masson Trichrome and Picrosirius Red Staining

Masson trichrome and Picrosirius Red staining were performed on lung and liver sections to visualize collagen-associated extracellular matrix alterations after polytrauma [34]. Because the observation period was limited to 7 d, these staining changes were interpreted as early extracellular matrix remodeling rather than established fibrosis. Masson trichrome and Picrosirius Red staining were evaluated qualitatively. No quantitative collagen-positive area or fibrosis score was calculated in the present study.

### 2.15. Statistical Analysis

Statistical analyses were performed using GraphPad Prism 8.0.2 (GraphPad Software, San Diego, CA, USA). Data are presented as mean ± SD. Longitudinal mNSS, rotarod, and open-field outcomes obtained repeatedly from the same animals were analyzed using two-way repeated-measures ANOVA, with injury group as the between-subject factor and time as the within-subject factor. The sphericity assumption was evaluated, and the Greenhouse–Geisser correction was applied when appropriate. Brain water content and ELISA measurements obtained from independent animals at each time point were analyzed using ordinary two-way ANOVA with injury group and time as fixed factors. Following the corresponding ANOVA, Sham-versus-polytrauma comparisons at individual time points were performed using Sidak’s multiple-comparisons test. Accordingly, the *p* values shown for these pairwise comparisons are adjusted for multiple testing. A two-sided *p* < 0.05 was considered statistically significant. Statistical significance is indicated as * *p* < 0.05, ** *p* < 0.01, and *** *p* < 0.001; ns indicates no significant difference.

## 3. Results

### 3.1. Polytrauma Induced Persistent Neurological, Motor, and Exploratory Dysfunction

Before injury, the longitudinally followed cohorts showed comparable neurological and rotarod performance. Polytrauma caused an immediate increase in mNSS, with the largest deficit observed at 0 h. Although mNSS values progressively declined over the subsequent 7 d, injured mice remained significantly impaired relative to Sham mice at every post-injury time point (Figure 1A). A similar pattern was observed in the rotarod test. Polytrauma markedly increased the number of falls during the 180-s test and reduced latency to fall throughout the observation period. Both measures improved progressively, but significant group differences remained at 7 d, indicating incomplete recovery of motor coordination and balance (Figure 1B,C).

Open-field trajectories further demonstrated marked restriction of spontaneous exploration after polytrauma (Figure 1D). Total distance traveled was substantially lower in injured mice at all examined time points and gradually increased over the 7-d period, but did not return to Sham levels (Figure 1E). Time spent in the center and the number of center entries were also reduced after polytrauma, with only partial recovery during the later phase (Figure 1F,G). Collectively, these results demonstrate sustained neurological, motor, locomotor, and exploratory dysfunction extending from the immediate post-injury period through day 7.

### 3.2. Polytrauma Produced Time-Dependent Brain, Liver, and Lung Histopathological Injury

**Figure 2:**
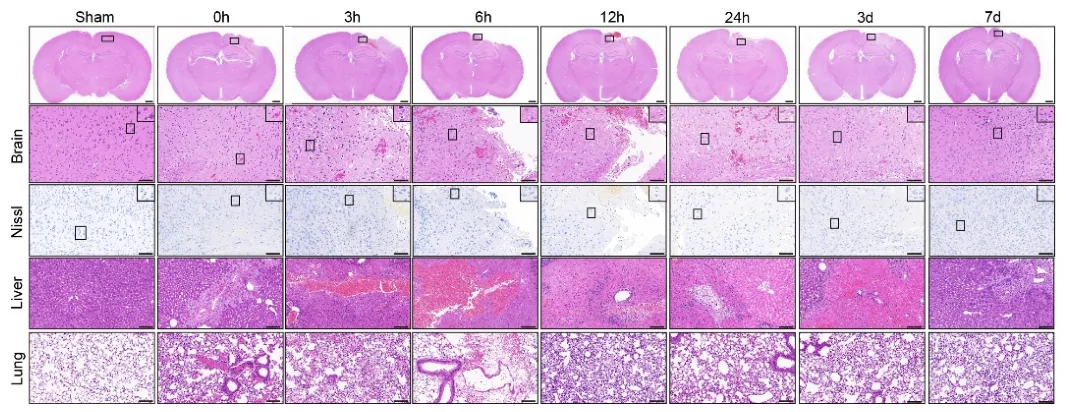
Time-dependent brain, liver, and lung histopathological alterations after experimental polytrauma. Representative histological images from one common Sham histology cohort and polytrauma mice at 0 h, 3 h, 6 h, 12 h, 24 h, 3 d, and 7 d. The same Sham cohort was used as the common histological reference for all post-injury time points; therefore, the Sham panels do not represent seven independent time-matched Sham cohorts. Rows show whole-coronal brain HE staining, higher-magnification HE staining of the injured brain region, Nissl staining of the brain injury region, liver HE staining, and lung HE staining. Scale bars, 50 μm.

Using the single Sham histology cohort as the common reference, qualitative examination of representative histological sections revealed time-dependent tissue abnormalities in the brain, liver, and lung after polytrauma (Figure 2). In the brain, whole-coronal HE sections and higher-magnification images showed evolving focal tissue disruption within the injured hemisphere, accompanied by edema, altered cellular architecture, and regions of parenchymal disorganization. These changes were apparent during the acute phase and remained visible through the later post-injury time points. Nissl-stained sections showed reduced preservation of normal neuronal morphology and Nissl substance within the injured brain region relative to Sham tissue, consistent with neuronal structural injury. Peripheral organs also displayed time-dependent histopathological abnormalities. Liver HE sections showed disruption of normal hepatic architecture with variable sinusoidal congestion, hepatocellular injury, and focal inflammatory-cell infiltration across the post-injury period. Lung HE sections demonstrated alveolar architectural distortion, septal thickening, increased tissue cellularity, and focal peribronchial inflammatory-cell accumulation. Abnormalities remained evident during the subacute 3–7 d phase.

### 3.3. Lung and Liver Extracellular Matrix Staining Changed Dynamically During the 7-Day Post-Polytrauma Period

**Figure 3:**
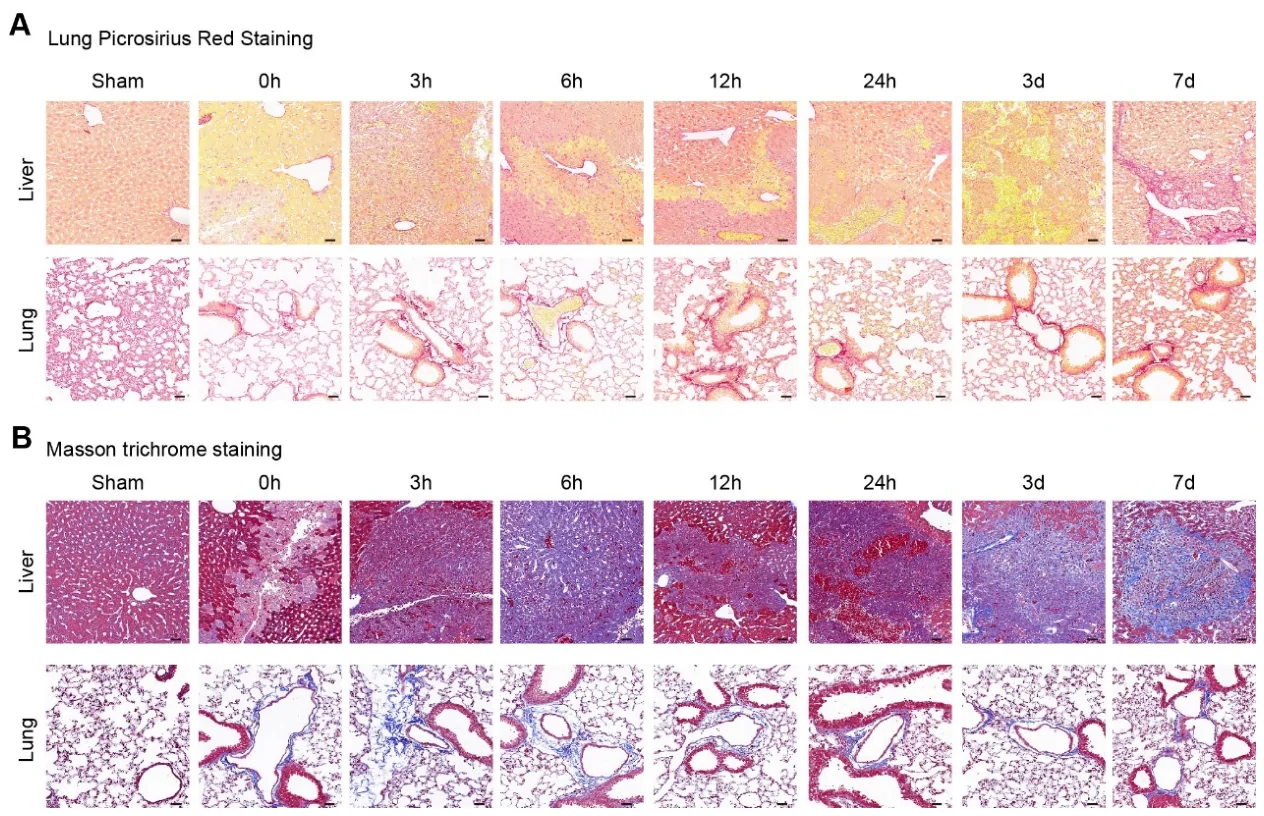
Dynamic collagen-associated extracellular matrix changes in liver and lung following polytrauma. (**A**) Representative Picrosirius Red-stained liver and lung sections from one common Sham histology cohort and polytrauma mice at 0 h, 3 h, 6 h, 12 h, 24 h, 3 d, and 7 d. (**B**) Representative Masson trichrome-stained liver and lung sections over the same time course. The same Sham cohort served as the common histological reference for all post-injury time points and should not be interpreted as separate time-matched Sham groups. These changes are interpreted as early extracellular matrix remodeling rather than established fibrosis. Scale bars, 50 μm.

Qualitative assessment of Picrosirius Red and Masson trichrome staining demonstrated dynamic collagen-associated extracellular matrix changes in both liver and lung following polytrauma (Figure 3). In the liver, collagen-associated staining became more conspicuous and spatially redistributed across the observation period, with prominent changes during later time points, including 3 and 7 d. Masson staining similarly showed increasing blue-stained extracellular matrix in selected regions during the subacute phase. In the lung, Picrosirius Red and Masson staining revealed time-dependent changes around airways, vessels and alveolar septal structures, with persistent extracellular matrix-associated staining through day 7. Given the short observation period, these findings are most appropriately interpreted as early extracellular matrix remodeling rather than established pulmonary or hepatic fibrosis. Because pulmonary and hepatic functional endpoints were not assessed in the present study, these histological changes should be interpreted as structural evidence of early extracellular matrix remodeling and not as direct evidence of impaired lung or liver function.

### 3.4. Polytrauma Caused Persistent Cerebral Edema but Temporally Dissociated Glucocorticoid and Noradrenergic Responses

**Figure 4:**
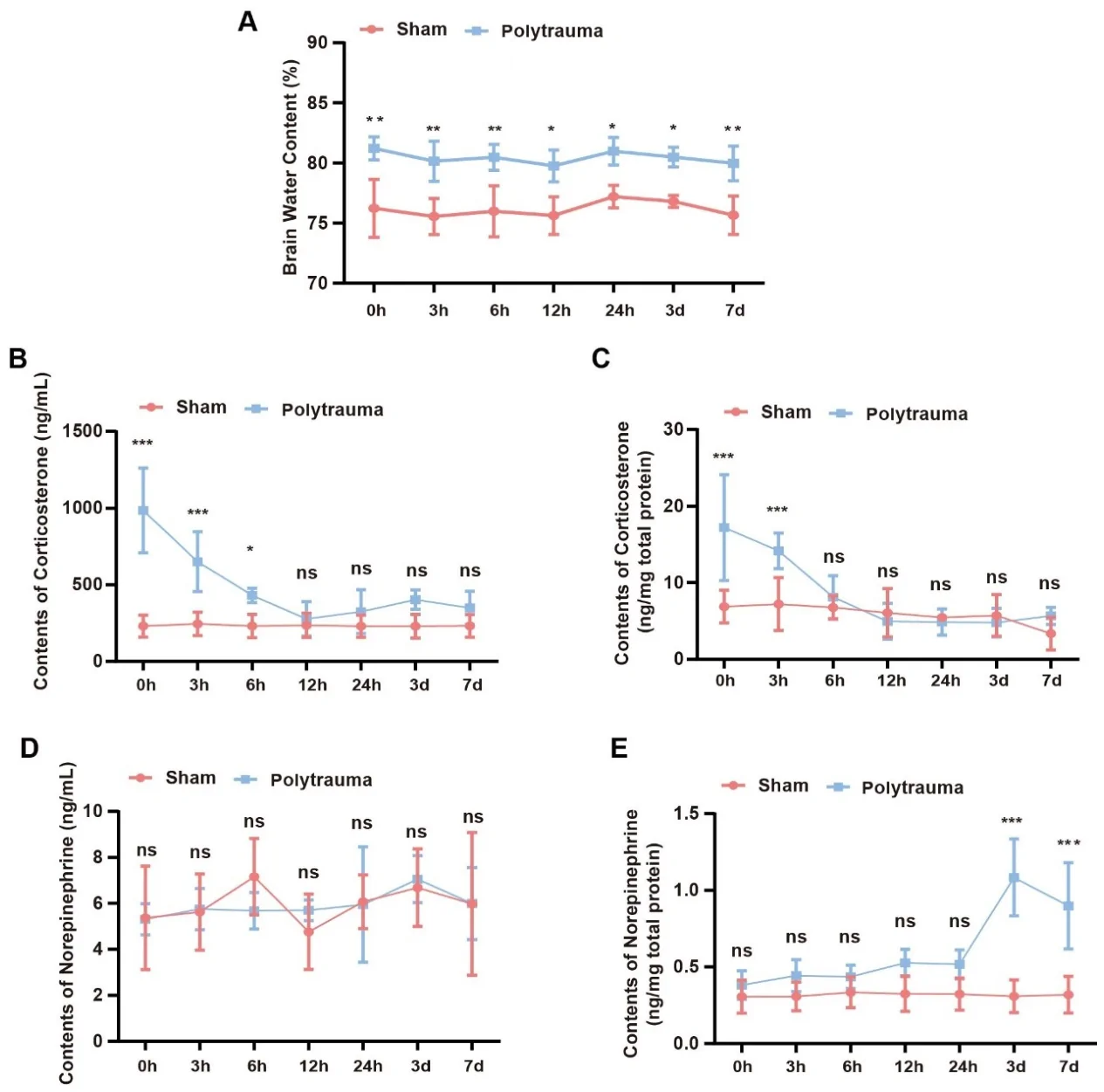
Persistent cerebral edema and temporally dissociated glucocorticoid and noradrenergic responses after polytrauma. (**A**) Brain water content. (**B**) Serum corticosterone concentration. (**C**) Corticosterone concentration in injured brain tissue. (**D**) Serum norepinephrine concentration. (**E**) Norepinephrine concentration in injured brain tissue. Brain water content: n = 3 mice per group at each time point. ELISA: n = 6 per group at each time point. Injured-brain concentrations were normalized to total protein. Data are mean ± SD. * *p* < 0.05, ** *p* < 0.01, *** *p* < 0.001; ns, not significant.

Brain water content was significantly higher in polytrauma mice than in Sham mice at all seven post-injury time points, indicating persistent cerebral edema from 0 h through 7 d (Figure 4A). In contrast to this sustained tissue-level abnormality, corticosterone showed a strongly transient response. Serum corticosterone was markedly increased at 0 h and 3 h and remained modestly but significantly elevated at 6 h; from 12 h onward, the difference between Sham and polytrauma groups was no longer significant (Figure 4B). Corticosterone in injured brain tissue was significantly elevated at 0 h and 3 h, but not at 6 h or later time points (Figure 4C).

Norepinephrine followed a distinct temporal profile. Serum norepinephrine did not differ significantly between groups at any time point during the 7-d period (Figure 4D). Injured-brain norepinephrine likewise remained comparable between groups from 0 h through 24 h, but increased sharply at 3 d and remained significantly elevated at 7 d (Figure 4E). Thus, the neuroendocrine data reveal a clear temporal dissociation: an immediate but short-lived glucocorticoid surge was followed by delayed local noradrenergic changes in injured brain tissue, while cerebral edema persisted throughout the entire observation period.

### 3.5. Systemic and Injured-Brain Pro-Inflammatory Responses Exhibited Distinct Temporal Profiles

**Figure 5:**
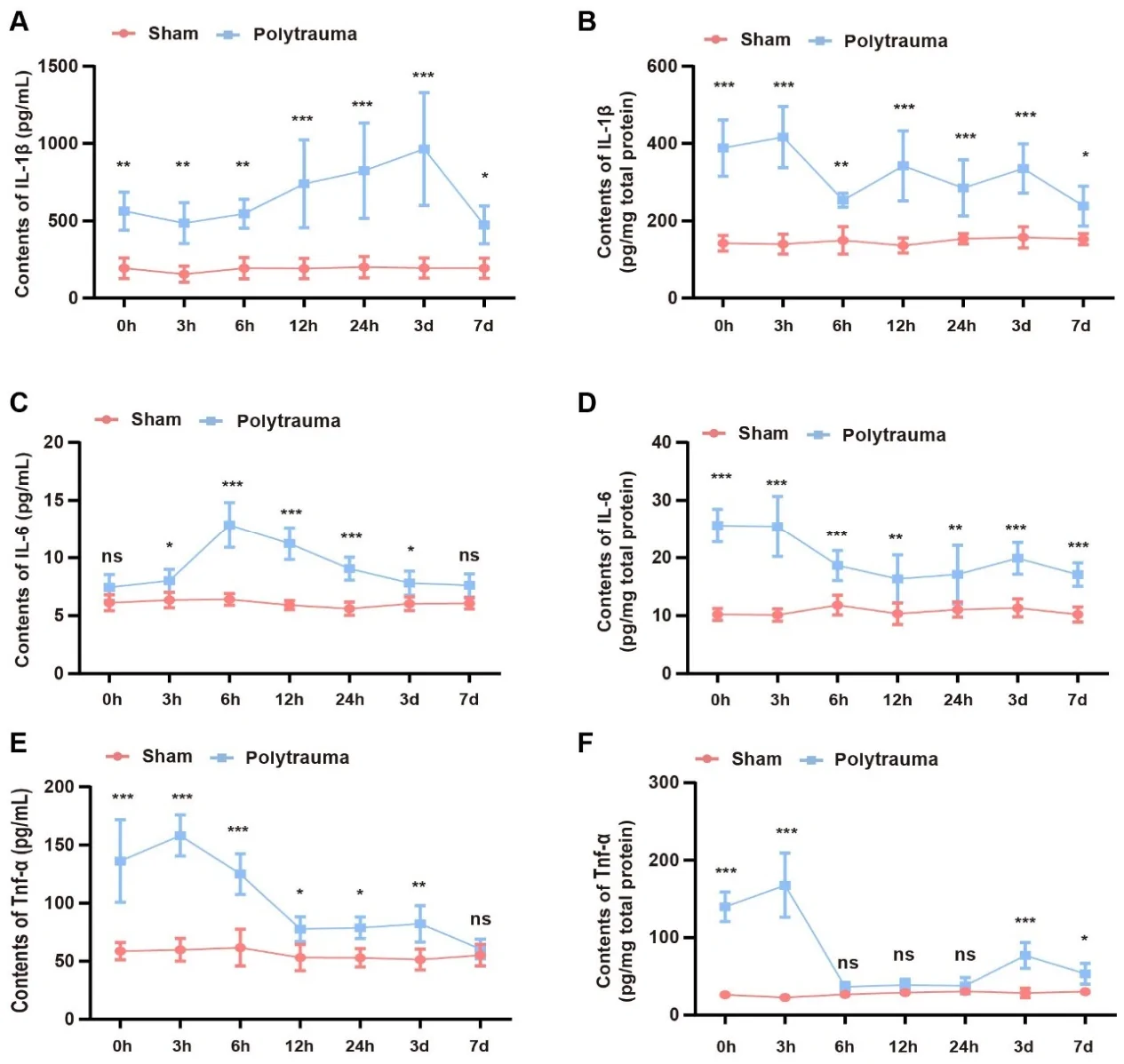
Systemic and injured-brain pro-inflammatory cytokines exhibit distinct temporal profiles following polytrauma. (**A**) Serum IL-1β. (**B**) IL-1β in injured brain tissue. (**C**) Serum IL-6. (**D**) IL-6 in injured brain tissue. (**E**) Serum TNF-α. (**F**) TNF-α in injured brain tissue. ELISA: n = 6 per group at each time point. Brain-tissue values were normalized to total protein. Data are mean ± SD. * *p* < 0.05, ** *p* < 0.01, *** *p* < 0.001; ns—not significant.

Polytrauma induced robust but cytokine-specific changes in systemic and injured-brain pro-inflammatory mediators (Figure 5). Serum IL-1β was significantly elevated at every post-injury time point. Levels were already increased at 0 h, remained elevated through 24 h, reached the highest mean level at approximately 3 d, and remained above Sham values at 7 d (Figure 5A). IL-1β in injured brain tissue was also significantly elevated at all time points, showing strong early increases at 0–3 h, a transient decrease at 6 h and renewed elevations during the 12 h–3 d period before declining at 7 d while remaining significantly above Sham (Figure 5B). The persistence of IL-1β in both compartments contrasted with the more restricted systemic profiles of IL-6 and TNF-α.

Serum IL-6 was not significantly different at 0 h, increased at 3 h, peaked at 6 h, remained significantly elevated at 12 h and 24 h, and was still modestly increased at 3 d before returning to a nonsignificant difference by 7 d (Figure 5C). In contrast, IL-6 in injured brain tissue was significantly elevated at every time point from 0 h through 7 d, with the highest levels during the early 0–3 h phase and persistent secondary elevations thereafter (Figure 5D). These findings indicate that local cerebral IL-6 dysregulation outlasted the systemic IL-6 response.

Serum TNF-α increased immediately after polytrauma, reached its highest mean level at approximately 3 h, remained significantly elevated through 3 d, and was no longer significantly different from Sham at 7 d (Figure 5E). Injured-brain TNF-α displayed a biphasic pattern: marked increases were present at 0 h and 3 h, values approached Sham levels at 6–24 h and a secondary increase emerged at 3 d and remained detectable at 7 d (Figure 5F). Together, these data demonstrate that systemic and cerebral inflammation are temporally coupled during some phases but dissociate during others and that no single cytokine adequately represents the post-polytrauma inflammatory trajectory.

### 3.6. Counter-Regulatory Cytokines Were Activated in Distinct Systemic and Cerebral Time Windows

**Figure 6:**
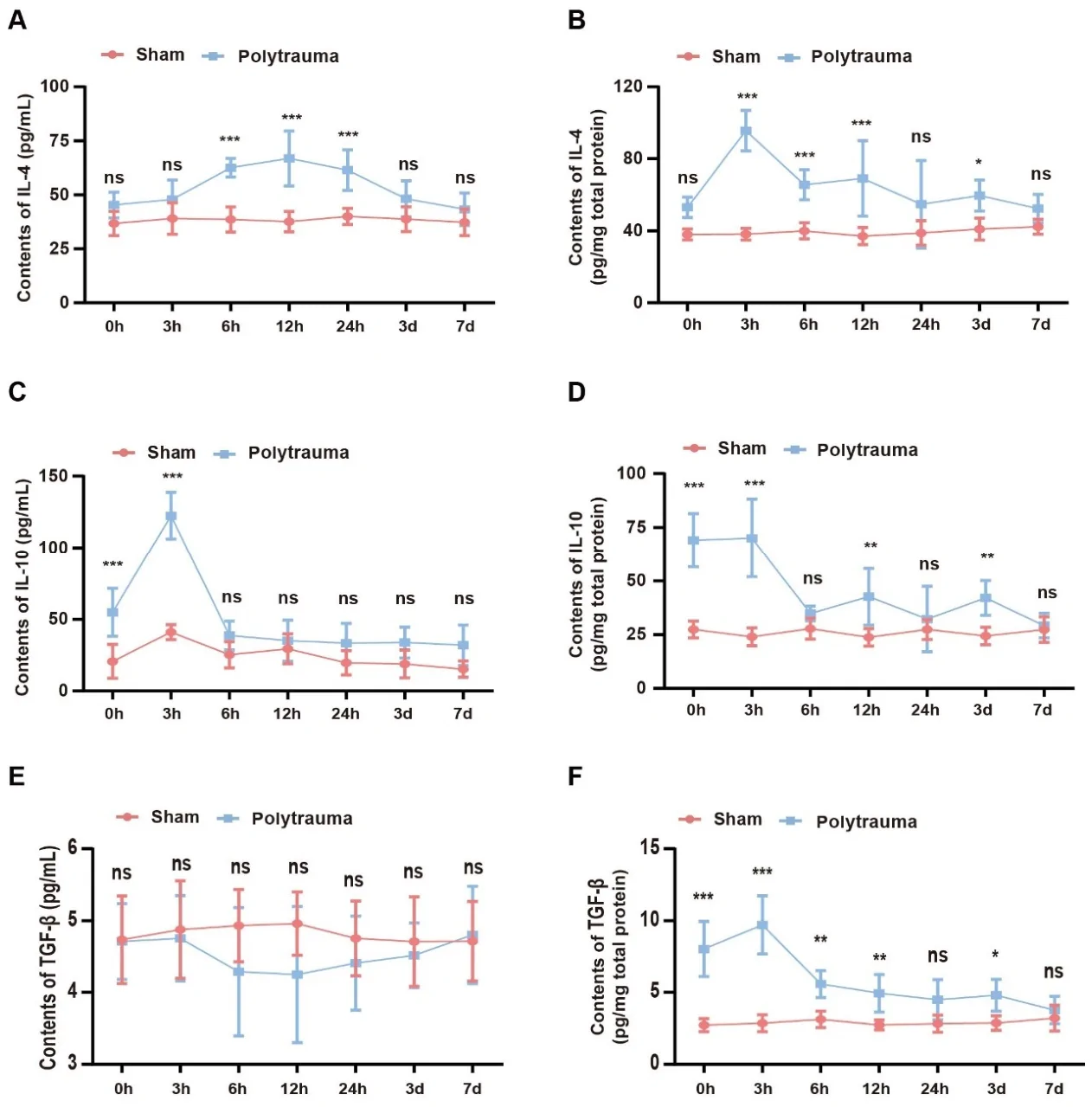
Counter-regulatory cytokine responses in serum and injured brain tissue following polytrauma. (**A**) Serum IL-4. (**B**) IL-4 in injured brain tissue. (**C**) Serum IL-10. (**D**) IL-10 in injured brain tissue. (**E**) Serum TGF-β. (**F**) TGF-β in injured brain tissue. ELISA: n = 6 per group at each time point. Brain-tissue values were normalized to total protein. Data are mean ± SD. * *p* < 0.05, ** *p* < 0.01, *** *p* < 0.001; ns—not significant.

The pro-inflammatory response was accompanied by temporally heterogeneous changes in IL-4, IL-10 and TGF-β (Figure 6). Serum IL-4 remained comparable to Sham at 0 and 3 h, increased significantly at 6 h, 12 h and 24 h, and returned toward Sham values by 3–7 d (Figure 6A). IL-4 in injured brain tissue showed an earlier response, with a pronounced increase at 3 h that remained significant at 6 and 12 h. The difference was not significant at 24 h, a smaller secondary increase was present at 3 d, and levels were no longer different at 7 d (Figure 6B).

Serum IL-10 displayed a rapid early response, increasing significantly at 0 h and peaking at 3 h before returning to values that were not significantly different from Sham from 6 h onward (Figure 6C). Injured-brain IL-10 was also elevated at 0 and 3 h, normalized at 6 h, increased again at 12 h, returned toward Sham at 24 h, and showed another significant increase at 3 d before normalizing by 7 d (Figure 6D). This local pattern suggests a more complex multiphasic immune-regulatory response within injured brain tissue than in the circulation.

Serum TGF-β remained unchanged throughout the 7-d observation period (Figure 6E). In contrast, TGF-β in injured brain tissue was significantly elevated at 0 and 3 h, remained increased at 6 and 12 h, was not significantly different at 24 h, showed a smaller elevation at 3 d, and returned toward Sham levels by 7 d (Figure 6F). Overall, the regulatory cytokine data demonstrate that counter-regulatory signaling occurs concurrently with pro-inflammatory activation but follows mediator- and compartment-specific temporal trajectories.

## 4. Discussion

The present study provides an integrated temporal characterization of neurological dysfunction, neuroendocrine stress, systemic and cerebral inflammation, and multiorgan pathological changes during the first 7 days after experimental head–thorax–abdomen polytrauma. The principal finding is that these responses do not evolve synchronously. Instead, the data support a multistage response in which an immediate glucocorticoid surge is superimposed on persistent neurological impairment and cerebral edema, followed by prolonged and cytokine-specific inflammatory abnormalities, delayed injured-brain norepinephrine elevation, and evolving pathological remodeling in the brain, lung and liver. This temporal dissociation is the central feature of the current dataset and provides a framework for understanding why a single biomarker or single sampling time is unlikely to capture the biological complexity of polytrauma.

Neurological dysfunction was both immediate and persistent. mNSS, rotarod performance and open-field behavior were markedly altered after injury, and although all showed gradual improvement, none fully normalized by day 7. The behavioral trajectory therefore differed from that of the early glucocorticoid response. Brain water content was also elevated at every post-injury time point, indicating that cerebral edema or altered tissue water homeostasis persisted after corticosterone had returned toward Sham levels. The representative HE and Nissl images provide a structural correlate for this functional impairment by demonstrating sustained abnormalities in the injured brain region, consistent with the prolonged secondary injury and neuroinflammatory processes described after TBI [11,17,18,23].

The neuroendocrine findings reveal one of the clearest examples of temporal dissociation. Serum corticosterone increased dramatically immediately after polytrauma and remained elevated through 6 h, but the group difference disappeared by 12 h. Injured-brain corticosterone normalized even earlier, with significant differences confined to 0–3 h. This rapid normalization contrasts with persistent cerebral edema, behavioral dysfunction and inflammatory activation. Accordingly, normalization of glucocorticoid concentrations should not be interpreted as resolution of the overall post-traumatic state. Rather, corticosterone appears to define a narrow early phase of the response, consistent with the rapid dynamics of the HPA-axis stress response [7,10,35].

Norepinephrine showed the opposite temporal behavior. Circulating concentrations remained unchanged throughout the observation period, whereas injured-brain norepinephrine increased only during the later 3–7 d phase. The absence of a systemic change together with a delayed local increase indicates that serum norepinephrine does not necessarily reflect catecholaminergic alterations within injured brain tissue [8,9,11,35]. The current study did not directly measure sympathetic nerve activity, central noradrenergic neuronal firing, catecholamine turnover, hypothalamic–pituitary–adrenal axis activity, ACTH, or CRH. Therefore, these data should be interpreted as evidence of temporally distinct neuroendocrine mediator profiles rather than proof of sequential activation of the HPA axis and sympathetic nervous system.

The inflammatory data further demonstrate that both mediator identity and tissue compartment determine the temporal response. IL-1β was the most persistent pro-inflammatory mediator, remaining elevated in serum and injured brain tissue throughout the 7-d study. This sustained profile differs from the relatively transient serum IL-6 response, which peaked around 6 h and approached Sham levels by day 7 and from serum TNF-α, which was maximal early and no longer significantly elevated at 7 d. In the brain, however, IL-6 remained increased throughout the observation period, and TNF-α showed a biphasic pattern with strong 0–3 h elevations and a secondary 3–7 d increase. These differences support the concept that systemic inflammatory measurements alone cannot fully represent ongoing neuroinflammation after polytrauma [14,16,17,18,19,21].

Importantly, the paired serum and injured-brain measurements demonstrated that inflammatory regulation after polytrauma was both mediator- and compartment-dependent. Systemic IL-6 and TNF-α responses largely subsided during the later phase, whereas selected cytokine abnormalities persisted or re-emerged in injured brain tissue. Regulatory mediators also followed distinct temporal patterns in the two compartments. These findings emphasize that circulating inflammatory markers do not necessarily reflect the ongoing inflammatory state within injured brain tissue and support the use of compartment-specific and time-resolved measurements after polytrauma. Accordingly, the present cytokine profiles should be interpreted as temporal markers of inflammatory regulation rather than as a comprehensive assessment of all upstream innate immune mechanisms.

The histopathological findings extend the temporal response beyond the brain. Lung and liver HE images demonstrated structural injury across acute and subacute phases, while Picrosirius Red and Masson trichrome staining showed evolving collagen-associated extracellular matrix alterations. These observations indicate that peripheral organ consequences persist after the earliest stress response has subsided. Because the observation period was limited to 7 d, the collagen-associated changes should not be labeled established fibrosis. The more conservative interpretation is early extracellular matrix remodeling, reflecting tissue repair, stromal reorganization, or evolving injury-associated matrix deposition [34,36,37,38,39,40,41]. In addition, the histopathological analyses were primarily qualitative, and quantitative histological scoring, morphometric analysis, and collagen-positive area measurements were not performed. These approaches will be important for quantitatively defining the magnitude and progression of brain and peripheral-organ pathology in future studies.

The functional significance of these extracellular matrix alterations remains unknown. The current study did not include pulmonary mechanics, gas-exchange measurements, serum liver enzymes, or hepatic synthetic-function assays. Accordingly, the observed collagen-associated changes cannot be directly linked to organ dysfunction. Future studies integrating quantitative morphometry with organ-specific physiological and biochemical measurements will be needed to determine whether early structural remodeling predicts subsequent functional impairment.

Taken together, the data support three partially overlapping temporal phases. During the first 0–6 h, polytrauma is characterized by severe neurological dysfunction, cerebral edema, a pronounced glucocorticoid surge, rapid systemic inflammatory activation, and strong local inflammatory responses. During the 12–24 h intermediate phase, glucocorticoid concentrations largely normalize, whereas neurological abnormalities, cerebral edema, IL-1β, injured-brain IL-6, and several regulatory cytokines remain abnormal. During the 3–7 d subacute phase, functional recovery remains incomplete, injured-brain norepinephrine rises, IL-1β persists, selected local cytokines show secondary elevations, and extracellular matrix remodeling becomes increasingly prominent. This organization emphasizes that the dominant biological targets after polytrauma change over time.

This temporal framework may have therapeutic implications. Interventions directed at the initial neuroendocrine stress response would likely require administration during a narrow early window. In contrast, strategies targeting persistent neuroinflammation, cerebral edema, secondary cytokine activation, or tissue remodeling may remain relevant for longer periods [18,21,22,42]. The current study was not designed to test treatment efficacy or causal relationships, so these therapeutic implications remain hypothesis-generating. Nevertheless, the data provide a rational basis for phase-specific intervention studies rather than assuming that one therapeutic target is equally relevant throughout the post-traumatic course.

## 5. Limitations and Future Perspectives

Several limitations should be considered. The present experiments were performed exclusively in young male mice aged 6–8 weeks. Because age can substantially influence neuroendocrine activation, inflammatory responses, vascular physiology, tissue repair, and neurological recovery after trauma, the current results should not be directly extrapolated to mature or aged populations. Future studies incorporating mature adult and aged animals will be necessary to determine whether the temporal response patterns identified here are age-dependent [43]. Second, the observation period ended at 7 d and therefore does not address chronic neurological recovery or mature organ fibrosis. Third, corticosterone and norepinephrine were measured as neuroendocrine mediators, but upstream HPA-axis regulators and direct sympathetic activity were not assessed; mechanistic conclusions regarding specific neuroendocrine pathways are therefore not warranted. Fourth, cytokine measurements demonstrate temporal associations but do not establish causality between specific mediators and neurological or organ injury. Finally, the combined model does not isolate the relative contribution of brain, thoracic, and hepatic injury to each systemic response, and future studies using component-injury control groups could address this question. In addition, several important components of trauma-associated innate immune and hematological responses were not examined in the present study. In particular, coagulation abnormalities, platelet dysfunction, fibrinolytic alterations, complement activation, and upstream damage-associated molecular patterns were not directly assessed. HMGB1 is an important endogenous DAMP that may link tissue injury to innate immune and coagulation responses after trauma. Future studies integrating HMGB1 localization and release with coagulation, fibrinolysis, complement activation, and cytokine dynamics will be required to determine how these interacting systems contribute to the temporal evolution of polytrauma.

Despite these limitations, the integration of longitudinal functional measurements with paired systemic and injured-brain biochemical analysis and multiorgan histopathology provides a broad view of the acute-to-subacute evolution of experimental polytrauma. The strong temporal mismatch among glucocorticoid stress, local norepinephrine, inflammatory mediators, neurological deficits and tissue remodeling is a notable feature of the study and supports the use of time-resolved experimental designs in future polytrauma research.

## 6. Conclusions

Experimental head–thorax–abdomen polytrauma induces a temporally heterogeneous response involving persistent neurological dysfunction and cerebral edema, a rapid but transient glucocorticoid surge, prolonged but cytokine-specific systemic and cerebral inflammation, delayed injured-brain norepinephrine elevation, and evolving brain, lung, and liver pathology with early extracellular matrix remodeling. These biological processes do not normalize simultaneously. Recognition of their distinct temporal trajectories may help identify phase-specific biomarkers and guide the timing of future mechanistic and therapeutic studies after severe polytrauma.

## Data Availability

The original contributions presented in the study are included in the article. Further inquiries can be directed to the corresponding authors.
